# Intraocular Pressure Measurements in Standing, Sitting, and Supine Position: Comparison between Tono-Pen Avia and Icare Pro Tonometers

**DOI:** 10.3390/jcm11216234

**Published:** 2022-10-22

**Authors:** Maddalena De Bernardo, Giulia Abbinante, Maria Borrelli, Margherita Di Stasi, Ferdinando Cione, Nicola Rosa

**Affiliations:** 1 Department of Medicine, Surgery and Dentistry, Scuola Medica Salernitana, University of Salerno, 84131 Salerno, Italy; 2 Department of Ophthalmology, Heinrich Heine University, 40210 Düsseldorf, Germany

**Keywords:** intraocular pressure, Icare Pro, standing position, supine position, Tono-Pen Avia

## Abstract

Background: Intraocular pressure (IOP) is influenced by body position. The purpose of this study is to compare the IOP measurements obtained with two different devices, to investigate IOP changes in standing, sitting, and supine positions. Methods: In this comparative prospective case series, IOP was measured in sitting, supine, prone, and standing (standing 1) positions and again five minutes after standing (standing 2), utilizing an Icare Pro (ICP) and a Tono-Pen Avia (TPA) in the 64 eyes of 32 healthy subjects. Results: Compared to the sitting position, both devices showed an increase in the IOP both in supine and standing 2 positions (*p* < 0.05). The mean IOP difference between the two devices was: in the sitting position, 0.57 ± 2.10 mmHg (range: −3.80 to 6.60 mmHg) (*p* < 0.05), in the supine position, 0.93 ± 2.49 mmHg (range: −4.50 to 7.10 mmHg) (*p* < 0.05), in the standing 1 position, 0.37 ± 1.96 mmHg (range: −5.20 to 5.00 mmHg) (*p* = 0.102), and in the standing 2 position 0.73 ± 2.03 mmHg (range: −4.5 to 6.4 mmHg) (*p* < 0.001). Conclusions: The results highlight an agreement between the TPA and ICP, both confirming not only the increase in IOP in the supine position, but also showing an increase in the standing 2 position. Therefore, it is suggested to perform such measurements in patients with glaucoma, to explain its progression in an apparently normal tension or in compensated patients.

## 1. Introduction

During an ophthalmic examination, intraocular pressure (IOP) is routinely measured as it is a well-known risk factor for glaucoma onset and progression. [1,2,3]. A single measurement is not sufficient to rule out the development of this condition, as the IOP has circadian variations that can influence the progression of glaucoma [4,5,6]. The body position was suggested as one of the causes of this fluctuation; in fact, several studies have shown that the IOP could significantly rise when a person is lying down compared to when they are in the sitting position, which could explain some nocturnal elevations [7,8]. During the day, several people spend hours in the standing position, so it might be useful to know if a change in IOP occurs in this situation. In a previous paper, utilizing an I Care Pro (ICP) Tonometer, we were able to prove that both in the supine and in the standing position, there was an increase in IOP [9], but we were not able to find other studies that reported a comparison with other instruments, that confirmed such a finding in standing position. The IOP measurements are mainly performed with Goldmann applanation tonometry (GAT), which is nowadays considered the gold standard for these measurements, but this device can be used only with the patient seated at a slit lamp [10]. Some prototypes of GAT have been designed to be used in the reclining position [11], but these are not available on the market. On the contrary, there are several hand-held IOP measuring devices that can be used in a non-seated position.

The purpose of this study was to compare IOP measurements obtained with different devices, to check if they both confirmed these findings and their comparability. For such a purpose, we decided to compare two devices: Icare PRO (ICP) and Tono-Pen AVIA (TPA).

## 2. Materials and Methods

### 2.1. Selection of Patients

The study was carried out in adherence to tenets of the World Medical Association’s Declaration of Helsinki, Institutional Review Board (IRB) approval was obtained (Cometico Campania Sud, Italy, prot. n° 16544), and an informed consent was achieved from all participants included in the study. Each subject underwent a general physical checkup, demonstrating the absence of any systemic disease. Patients with systemic and ocular diseases which could potentially interfere with the purpose of the study, such as diabetes, collagenopathies, dry eyes, keratoconus, uveitis, corneal and lens opacities, glaucoma, previous intraocular or corneal surgery, pregnancy, and an inability to fixate on the target, were excluded from the study. All participants underwent a complete ophthalmological examination, including postoperative corrected distance visual acuity, a refractive error, and a fundus examination. Moreover, all the patients did not have a smoking history or an alcohol addiction.

### 2.2. IOP Measurement

The IOP measurement was performed in the time slot between 10 am and 12 pm by two different observers, who were not aware of the results obtained from the other. The measurements were obtained in the following order: the sitting position, supine after five minutes of lying down, standing (standing 1), and five minutes after the patient was staying in the standing position (standing 2).

IOP was measured with the ICP (Icare Finland Oy, Finland version 1.1) and then with the TPA (Reichert Inc., Depew, NY, USA). Although the ICP does not require anesthesia, before the first measurement, both eyes were anesthetized with oxybuprocaine eye drops, to avoid differences in performing the two measurements, even if it has been shown that oxybuprocaine does not change corneal thickness [12].

The ICP measures the IOP, converting the acceleration of a small probe pushed against the cornea [13]. This procedure does not require local anesthesia or calibration, due to a very brief contact with the corneal surface.

The ICP provides the mean IOP from six consecutive tests, showing the accuracy by a color code shown below the value of the IOP. In case of being within the normal limits of variance, the indicator is green; if the variance is greater than normal, it is yellow; and if the variation is unacceptably high, it is red. In these two last cases, the measurements should be carefully considered [11].

Measurements were accepted only when the indicator was green, otherwise they were repeated.

The TPA uses the same physical principle as the GAT to measure the IOP, but the applanated area is much smaller, since the transducer tip, covered with a new latex tip cover before each measurement, has a diameter of 1.0 mm.

The mean IOP readings were automatically averaged by the instrument, when ten valid readings were obtained, by lightly touching the central cornea. The measurement was shown on the liquid crystal display, which is situated on the side of the device, and together it displayed the “statistical confidence indicator”, indicating that the standard deviation of the valid measurements is 5% or less of the number shown. The higher the value, the more reliable the measurement is. Only values higher than 90 were accepted.

### 2.3. Central Corneal Thickness and Axial Eye Length

To check if potential differences were related to some ocular parameters, such as the central corneal thickness (CCT) or axial eye length (AL), the patients were examined with a Pentacam HR (Oculus, Wetzlar, Germany, version 1.19r11) and an IOLMaster (Zeiss, Jena, Germany, version 5.4.4.00006) [14,15].

### 2.4. Statistical Analysis

The exact Kolmogorov–Smirnov test showed a normal distribution (*p* > 0.05) for all data. For this reason, a paired *t*-test was used for all statistical evaluations. Moreover, Bland–Altman plots were also performed. A p-value less than 0.05 was considered statistically significant.

A correlation between the TCP and TPA measurements in all position were evaluated by Pearsons’s correlation coefficient (r). In addition, a correlation between the CCT and the difference between the ICP and TPA in all positions, and between the AL and the difference between the ICP and TPA in all positions, were evaluated by the same test. The required sample size was calculated with G*Power software (Version 3.1.9.7, Faul, Erdfelder, Lang, and Buchner, 2020, Available at https://www.gpower.hhu.de (accessed on 9 July 2022)). It was estimated that with a significance level of 5% and a test power of 80%, a sample size of 64 eyes would be necessary to detect a difference in the mean error of 0.50 mmHg, given a within-subject standard deviation (SD) for IOP equal to 1.50 mmHg.

## 3. Results

### 3.1. Patient Characteristics

Sixty-four eyes of 32 patients (16 males) aged between 21 and 55 years (mean 29 ± 9 years) and with a refractive error, in terms of being spherically equivalent, between −6.63 and +1 D (mean: −2.32 ± 2.19 D), were included in this comparative prospective observational study.

### 3.2. Comparison between TPA and ICP IOP Measurements

Table 1 summarizes the IOP measurements obtained by the two instruments in the different positions, showing that there were statistically significant differences between the IOP measurements obtained by the same device (all *p* < 0.05), except for the difference in IOP obtained by the TPA between the sitting and standing 1 position (*p* = 0.187).

**Figure 1:**
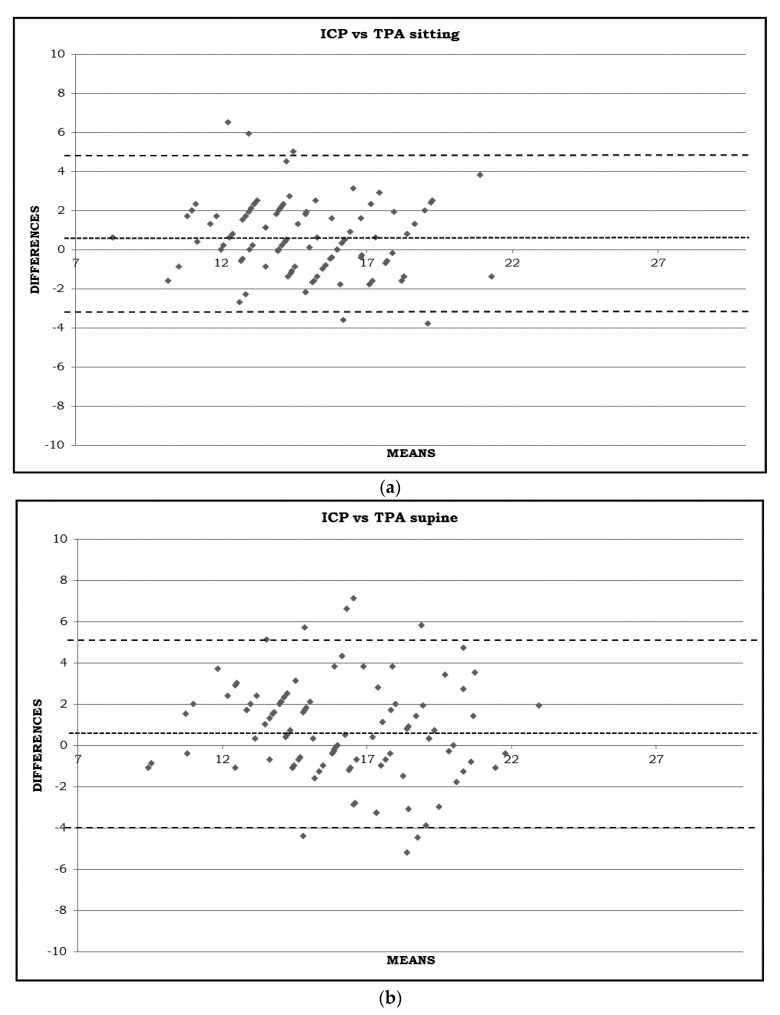

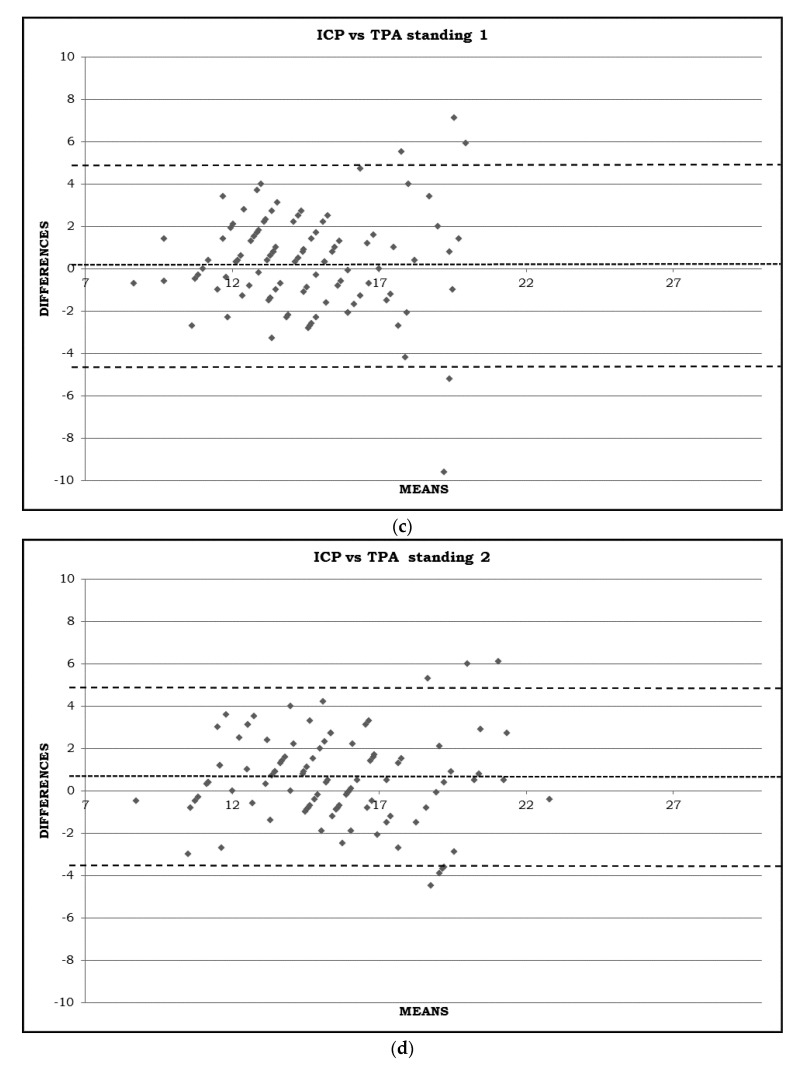
(**a**) IOP measured in sitting position using Icare PRO (ICP) and Tono-Pen AVIA (TPA) (mmHg). Bland–Altman diagram with mean difference and agreement limits (including 95% of all difference values). (**b**) IOP measured in supine position using Icare PRO (ICP) and Tono-Pen AVIA (TPA) (mmHg). Bland–Altman diagram with mean difference and agreement limits (including 95% of all difference values). (**c**) IOP measured in standing 1 position using Icare PRO (ICP) and Tono-Pen AVIA (TPA) (mmHg). Bland–Altman diagram with mean difference and agreement limits (including 95% of all difference values). (**d**) IOP measured in standing 2 position using Icare PRO (ICP) and Tono-Pen AVIA (TPA) (mmHg). Bland–Altman diagram with mean difference and agreement limits (including 95% of all difference values).

The IOP is higher in the standing 2 and supine position than in the sitting one, both with the ICP and TPA (all *p* < 0.05). It is interesting to note that, switching from supine to standing 1, there is a significant decrease in IOP (*p* < 0.001), although such a decrease is not significant when compared to the sitting one with the TPA (*p* = 0.187). Lastly, after 5 min standing, the IOP becomes higher than in the sitting position (*p* < 0.05) with both instruments. The comparison between the two devices indicated in Figure 1a–d and in Table 1 shows that, except in the standing 1 position, the measurements are significantly different, with the ICP values being higher than the TPA ones.

### 3.3. Correlation between TCP and TPA, Correlation between CCT, AL and IOP Measurements

There was a strong positive correlation between the measurements obtained by the ICP and TPA in all positions (all r ≥ 0.699, all *p* < 0.05). The CCT ranged from 470 to 598 µ (mean: 539.95 ± 30.36 µ) and the AL ranged from 21.70 to 27.16 mm (mean: 24.45 ± 1.29 mm). The correlations between the CCT and the difference between the ICP and TPA in all positions are shown in Table 2.

There was a weak negative, but not a statistically significant, correlation between the CCT and the difference between the two devices in all positions (all *p* > 0.05). The correlations between the AL and the difference between the ICP and TPA in all positions are shown in Table 2. In the same way, there was no correlation between the AL and the difference between the two devices in all positions (all *p* > 0.05).

## 4. Discussion

In a literary review on PubMed, we found few papers where the IOP was measured with different devices in the sitting and supine position, such as the TPA [11], ICP [16], and Tono-Pen [17,18,19,20,21], and even less comparing different the tonometers [11,22,23], but we were not able to find IOP measurements in the standing position in the references we studied.

Schweier et al. [11] measured IOP in the sitting position and then 10 min after in the reclining position, in the 36 eyes of 36 healthy people with the ICP, TPA, and GAT. They noticed that IOP in the sitting position was lower than the reclining one with both the handheld tonometers and the mean difference with the TPA (1.8 mmHg) being greater than with the ICP (0.8 mmHg).

Barkana et al. [22] tested, with the Tono-Pen XL and ICP, the IOP in the sitting position, and then after 10 min, tested it in the supine position, in 21 eyes of 21 healthy subjects. They observed a mean rise in the IOP of 0.9 mmHg from the sitting to the supine position with the Tono-Pen, but they found a mean decrease in 0.9 mmHg from the sitting to the supine position with the ICP, as compared to Schweier.

Lee et al. [24] compared the IOP measurements obtained using the Icare Pro rebound tonometer and Tono-Pen XL tonometer in the supine and right and left lateral decubitus body positions in the one hundred eyes of 50 healthy volunteers or glaucoma suspects. The IOP readings obtained using the Icare Pro and Tono-Pen showed good correlations in these two positions, although the Icare Pro readings were higher than the Tono-Pen readings in all of them.

Concerning the differences between the sitting and the supine position, our results confirm those obtained by Schweier [11] and Lee [24] but are in contrast with those obtained by Barkana [22]. Indeed, we found an increase in IOP with both the ICP and TPA, when changing the position from sitting to supine, even though we waited 5 min instead of 10.

Our results confirm also those ones of Nakakura et al. [23], who recently found an increase in IOP in the supine compared to in the sitting position using the ICP, Tono-Pen XL, and Kowa.

The present study confirms the results obtained with the ICP in a recently published paper by De Bernardo et al. Indeed, they observed a non-significant decrease in IOP from the sitting to the standing 1 position and a small but significant increase in IOP in the standing 2 position relative to the sitting position [9].

Several reasons could explain the increase in IOP in the supine position. Some authors suggest that there is a choroidal vascular engorgement caused by the redistribution of body fluids [25,26], while others suggest that there is an increase in the episcleral venous pressure [27,28,29].

Regarding the differences between the two devices (TPA e ICP) in the standing position, no comparison can be made within the international literature because we were not able to find similar papers.

Interestingly, both devices detected an IOP decrease immediately after standing, and an IOP rise in the standing position after five minutes.

The IOP rise in the standing position is slightly difficult to explain, as the IOP regulation’s exact mechanism is uncertain. It should be subject to systemic vascular monitoring, that could also explain the slight decrease when going from the supine to the standing position, that conversely becomes an increase 5 min after maintaining the standing position. [30,31,32,33].

The measurements obtained by the two devices (Tono-Pen Avia and ICare Pro) were different, but the detection of an increase and decrease was similar. The dissimilarity of the measures was not related to differences in the CCT or AL. However, our results confirm the importance of ensuring that measurements should be taken with the same instrument when monitoring ocular hypertension or glaucoma.

One criticism that could be made of this study is that we used the ICP and TPA, but not the GAT, which represents the gold standard in measuring IOP.

This criticism could be overcome because, even if the GAT is considered to be the gold standard, it is well known that several corneal parameters such as biomechanical properties [33,34], CCT [35,36,37,38], corneal irregularities [10], and refractive corneal surgery [38] can influence the GAT measurements. These limits could also be present with other tonometers, but the real problem is that to perform such measurements with the GAT in different positions is quite difficult, and as far as we know, no papers have considered it as a gold standard in the standing and supine positions too.

In addition, several papers compared the GAT with TPA or ICP in the sitting position [11,17,22]. Schweier et al. [11] founded a mean IOP measured by the GAT of 14.9 ± 3.5 mmHg, compared to a mean IOP measured by the ICP of 15.6 ± 3.1 mmHg, and with the TPA, of 14.8 ± 2.7 mmHg in the sitting position [11]. Barkana’s studies [17,22] showed, in the sitting position, a mean IOP for the GAT measurements lower than the Icare Pro and TonoPenXL measurements.

The findings show a slight agreement between the ICare Pro and Tono-Pen Avia.

Further studies in a larger group of patients comparing the IOP changes with parameters such as one’s age, gender, race (African ancestry), changes in blood pressure in different positions [27,28,29], nuclear sclerosis, iris color, myopia, EtOH use, and smoking could help in understanding the reasons of such an increase.

In order to better understand the development of glaucoma in apparently normal tension or in compensated patients, the IOP scale in the standing position along with the one in the supine position should be incorporated into clinical practice. In fact, certain occupations (e.g., waiters, policemen, and chefs) require a considerable amount of standing time, and our research not only confirms the increase in IOP in the supine position but, more importantly, indicates an increase in the standing position.

Future studies including glaucomatous patients could be helpful in terms of knowing whether the IOP fluctuations are greater with changes in the body’s position.

## Figures and Tables

**Table 1:** Comparison of intraocular pressure (IOP) measurement obtained in different positions both with Icare PRO (ICP) and Tono-Pen AVIA (TPA), with evaluation of difference of IOP measurements between ICP and TPA in each position. CI 95% = 95% confidence interval from the mean; SD = standard deviation; Min/Max = minimum and maximum measurement. *p* value = level of significance obtained by Paired T-test. Δ= Difference between ICP and TPA. All IOP parameters were reported in mmHg.

	Sitting			Supine			Standing 1			Standing 2		
	ICP	TPA	Δ	ICP	TPA	Δ	ICP	TPA	Δ	ICP	TPA	Δ
**Mean**	14.97	14.34	0.57	16.58	15.61	0.93	14.45	14.03	0.37	15.70	14.88	0.73
**CI 95%**	[14.30; 15.65]	[13.57; 15.12]	[0.03; 1.12]	[15.88; 17.30]	[14.75; 16.47]	[0.29; 1.58]	[13.83; 15.07]	[13.31; 14.75]	[−0.13; 0.88]	[14.94; 16.45]	[14.04; 15.71]	[0.20; 1.24]
**SD**	2.71	3.10	2.10	2.83	3.44	2.49	2.49	2.89	1.96	3.04	3.36	2.03
**Min**	8.60	8.00	−3.80	9.10	10.00	−4.50	8.30	9.00	−5.20	8.50	9.00	−4.50
**Max**	22.80	22.00	6.60	23.90	22.00	7.10	20.40	22.00	5.00	23.00	23.00	6.40
***p*** **value**	-	-	0.019	-	-	0.003	-	-	0.102	-	-	0.002

**Table 2:** Person’s correlation coefficient (r) between difference in intraocular pressure measurements obtained in different positions between Icare PRO (ICP) and Tono-Pen AVIA (TPA), and central corneal thickness (CCT) or axial length (AL).

	Difference between ICP and TPA			
	Sitting	Supine	Standing 1	Standing 2
**CCT**	−0.084	−0.084	−0.166	−0.083
**AL**	−0.182	−0.184	0.024	−0.018

## Data Availability

The data presented in this study are available on request from the corresponding author.
